# Application of Eutectic-Solvent-Based Liquid–Liquid Microextraction for Removal of Eight Bisphenols from Water and Industrial Samples

**DOI:** 10.3390/molecules31081357

**Published:** 2026-04-21

**Authors:** Michal Adámek, Petr Tůma, Zuzana Bosáková

**Affiliations:** 1 Department of Analytical Chemistry, Faculty of Science, Charles University, Albertov 8, 128 43 Prague, Czech Republic; michal.adamek@natur.cuni.cz; 2 Department of Hygiene, Third Faculty of Medicine, Charles University, Ruská 87, 100 00 Prague, Czech Republic

**Keywords:** bisphenol, eutectic solvent, liquid chromatography, mass spectrometry, solvent reuse, water purification

## Abstract

In this study, new types of eutectic solvents (ESs) are tested for their ability to remove the eight most common bisphenols (BPA, BPB, BPC, BPE, BPF, BPG, BPS, BPAP), which are environmentally monitored substances, from aqueous matrices. A total of 18 ESs based on hydrophobic organic acids, such as capric, caprylic, lauric, and myristic acids, and terpenes, such as DL-menthol, terpineol, linalool, and geraniol, are prepared and mixed in various molar ratios. The highest extraction yield for all types of BPs is achieved with a binary mixture of geraniol:caprylic acid prepared in a molar ratio of 1:1. This ES can be used repeatedly for five consecutive cycles achieving almost 100% recovery for BPB, BPC, BPG, and BPAP, while for BPA, BPE, and BPF, the yield drops to 97% and for BPS to 90%. The efficiency of ES extraction is verified using HPLC-MS/MS to determine the BPs in the aqueous phase. This is performed at a pentafluorophenylpropyl stationary phase with LOQs ranging from 0.24 to 29.1 ng/mL. The applicability of this HPLC-MS/MS method was demonstrated by monitoring the occurrence of BPs in thermal paper and other industrial samples.

## 1. Introduction

Bisphenols (BPs) are commonly used in the production of plastic containers, epoxy resins, food and beverage containers, water pipes, electronic devices, thermal paper, kitchen tableware, toys, and even dental fillings [1]. Due to the relatively good solubility of most BPs in water, undesirable contamination of the environment, drinking and utility water, food, beverages, etc., occurs. The human population is therefore exposed to the undesirable effects of BPs both directly from food and beverages and indirectly from the environment. The widespread use of BPs in plastic products has resulted in the contamination of human organisms with BPs even without intentional exposure. The most studied BP is bisphenol A (BPA), whose large-scale production for the manufacture of epoxy resins and polycarbonate began in the 1950s [2]. However, due to growing concerns about the safety of BPA [3], alternatives for the basic structure of BPs and the introduction of new functional groups that have a significant impact on physicochemical properties began to be considered (Table 1). Bisphenols B, F, S and AP were gradually introduced and became the most commonly used alternatives to BPA [4].

BPA and its analogs have the potential to cause mutagenic and carcinogenic effects in humans and impair endocrine function, acting as endocrine disruptors due to their similar structure to steroid hormones [5]. The effects of BPs are associated with a number of serious health disorders, such as obesity, diabetes mellitus, respiratory disorders, behavioral disorders, breast cancer, and developmental abnormalities in tooth structure. Numerous studies conducted on human populations have consistently revealed a significant association between environmental exposure to BPA and adverse health effects. Studies have shown that BPA can disrupt hormonal signaling pathways by binding to hormone receptors, altering hormone metabolism, or affecting hormone transport [6]. The best-known hormonal effect of most BPs is their estrogenic activity, where they act as agonists of the estrogenic receptor hERα, while their glucuronide metabolites lack this ability. BPB and BPC are stronger agonists of the hERα receptor than BPA [7]. The presence of a 4-hydroxyl group on the aromatic nucleus is considered the minimum structural requirement for estrogenic activity [8]. Hydrophobic substituents in the 1-methyl position on the central atom of the basic structure increase hormonal activity, while hydrophilic substituents such as hydroxymethyl or carboxyl groups decrease activity. In addition, a number of BPs have been shown to affect the function of ion channels, androgen, glucocorticoid, or thyroid hormone receptors; for a detailed overview, see the publication [9]. Observational clinical studies in humans have revealed that BPs cause an increase in arterial blood pressure, and have a negative effect on the lipid spectrum and ischemic heart disease [10]. Epidemiological studies also confirm the link between BPs and diseases such as female reproductive system cancer, reproductive dysfunction, and miscarriages, which can negatively affect conception and pregnancy maintenance [11]. BPs as endocrine disruptors can modify immune system cells or the way in which they produce inflammatory mediators [12].

The determination of BPs in food, environmental, and commercial samples is complicated by the matrix effect and therefore requires the use of an effective extraction technique. Conventional liquid–liquid extraction (LLE) consumes large volumes of solvents and is not always environmentally friendly. Therefore, it is replaced by liquid–liquid microextraction (LLME), where dichloromethane [13] or the more environmentally friendly octan-1-ol [14] is used to separate BPA from water. Dispersive liquid–liquid microextraction (DLLME) using a chlorinated extraction solvent and a dispersing agent (MeOH, EtOH, acetonitrile), followed by centrifugation, has become popular for environmental analysis [15,16]. Another variant of DLLME is a combination with ammonium sulfate salting-out, which has been used to extract BPs from canned coffee drinks [17]. Besides inorganic salt, the addition of monosaccharide was also employed to separate the organic and aqueous phases, and this approach has been used in the extraction of BPA and BPB from royal jelly [18] and beeswax [19]. In addition to BPs, an eucalyptol-based DLLME coupled to GC-MS was examined to determine selected phthalate esters in bottled water samples [20]. DLLME on molecularly imprinted polymers was also applied for simultaneous trace detection of estrogens in aqueous solution, followed by HPLC-MS/MS [21].

A more environmentally friendly alternative to conventional solvents is to use natural substances in the form of mixtures known as eutectic solvents (ES). ESs consist of a mixture of a hydrogen bond donor (HBD) and a hydrogen bond acceptor (HBA), between which a network of hydrogen bonds is formed. Due to the predominantly hydrophilic nature of ESs, their use for the extraction of pollutants from aqueous matrices is limited [22]. ESs based on thymol, camphor, undecylenic acid, and capric acid were prepared to remove polyaromatic hydrocarbons from water [23]. ESs composed of terpenes (menthol, terpineol, linalool, geraniol, and borneol) and organic acids ranging from formic to lauric acid were applied for the extraction of phytocannabinoids from natural cannabis materials [24].

Sensitive monitoring of BPs in aqueous matrices is typically performed using high-performance separation techniques such as high-performance liquid chromatography (HPLC), gas chromatography (GC), or capillary electrophoresis (CE), often in combination with mass spectrometry (MS). A HPLC MS/MS method with gradient elution and electrospray was developed to quantify nine BPs in wastewater [25]. Similar instrumentation was used to determine 30 BPs, including their analogs [26], and to screen for BPs in dairy products [27]. In addition to MS, BPs are also determined by HPLC coupled to UV detection at a wavelength of 210 nm with isocratic elution [28] or by HPLC combined with fluorescence with the addition of cyclodextrins to the mobile phase [29]. An electrochemical flow cell can also be used to detect BPs as phenolic substances [30]. A GC/MS method combined with salting-out LLE was developed to quantify 16 BP analogs in packaged foods and beverages [31]. BPA, as a phenol, can also be separated using CE in borate buffer and detected by UV [32,33,34].

The presented study focuses on LLME of eight of the most important BPs from aqueous matrices. Three different groups of ESs based on binary mixtures of organic acid/organic acid, terpene/organic acid, and menthol/organic acid are newly evaluated as extraction solvents, covering a wide range of BPs. A total of 18 different ESs are prepared and compared in terms of extraction efficiency, and the most effective ones are tested for the analysis of BPs in industrial samples. A sensitive HPLC-MS/MS methodology is developed for the determination of residual concentrations of eight BPs in aqueous matrices after their extraction with accurate identification of individual analytes based on product ion monitoring.

## 2. Results

### 2.1. Development and Evaluation of the HPLC-MS/MS Methodology

Standard solutions of BPA, BPB, BPC, BPE, BPF, BPG, BPS, BPAP, and IS at a concentration of 50 µg/mL were used to optimize tandem mass spectrometric detection. The injected volume was set at 10 µL. The mass analyzer was tested in both positive and negative ionization modes, and after comparing the responses, the negative mode was used for subsequent analyses. The desolvation gas (nitrogen) flow rate was set to 10 L/min at a temperature of 300 °C, the capillary voltage to 4000 V, the nebulizer pressure to 55 psi, and the transition time to 200 ms. During optimization, a mobile phase of 100% MeOH at a flow rate of 0.1 mL/min was used. BPs were first measured in scanning mode in the range of *m*/*z* 100 to 500. In the resulting spectra, the most intense ion signal was found for each analyte, and this ion was further used as a precursor ion. During single ion monitoring (SIM) mode, the voltage on the fragmentor had a range of 120–150 V to achieve the highest response. In product ion mode, the fragmentation of the precursor ion was monitored in the range of *m*/*z* 100 to 500. To achieve the highest signal intensity of the product ion, the collision energy was optimized in the range of 10 to 35 V. The peak with the second-highest intensity after the product ion peak was selected as the confirmation peak. The results of the tandem mass detection optimization are summarized in Table 2.

Although various types of columns with octadecyl stationary phase are most commonly mentioned in the literature for the separation of BPs [25,26,27,28,29,30,31,32,33], a pentafluorophenylpropyl (F5) column was chosen for this work. This column contains an electron-deficient phenyl group, which, due to the substitution of the benzene ring with fluorine atoms, provides π-π, weak steric and polar interactions. This stationary phase can be effectively used for the separation of basic, neutral, and acidic substances and offers alternative selectivity to C18. The Ascentis Express F5 column uses fused-core technology, which combines a solid core with a thin, porous layer of stationary phase on the surface.

**Figure 1:**
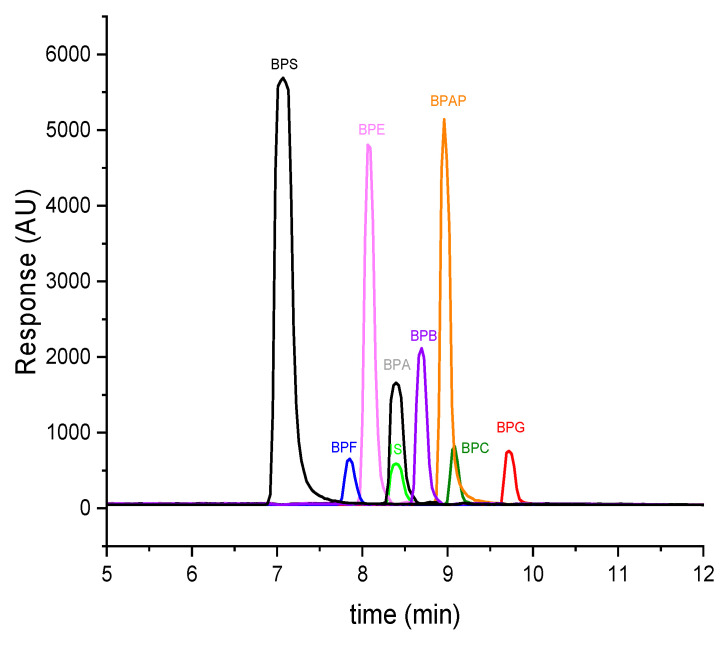
HPLC-MS/MS analysis of model mixture of bisphenols at 50 µg/mL concentration dissolved in DEI under optimized detection and separation conditions. AU—arbitrary unit, retention times: BPS (7.1 min), BPF (7.8 min), BPE (8.1 min), BPA (8.4 min), BPB (8.7 min), BPAP (9.0 min), BPC (9.1 min), BPG (9.7 min), and IS (8.4 min).

Firstly, isocratic elution was tested using MeOH and DEI in various volume ratios, but this approach was not suitable in terms of symmetry and peak focusing. Several gradient programs with different temperature profiles were then optimized, and the most suitable gradient option proved to be an initial MeOH/DEI ratio of 60:40 (*v*/*v*), increasing to 100% MeOH in 5 min and held isocratically to 11 min while thermostating the column at 45 °C. Under these conditions, BPs eluted at retention times from 7.1 min to 9.7 min, calibration curves were linear with *R*^2^ > 0.99, and LOQs ranged from 0.24 ng/mL to 29.1 ng/mL. Detailed information about the method is provided in Table 3. All samples were assayed in at least three replicates, and the resulting values are reported as the mean. MRM chromatograms of the individual BPs and IS are depicted in Figure 1.

### 2.2. Extraction Efficiency of ESs for LLE of BPs from Deionized Water

#### 2.2.1. ES Based on a Mixture of Two Carboxylic Acids

LLE was performed with an equimolar mixture of 8 BPs dissolved in DEI at three concentration levels of 10, 20, or 40 µg/mL. In the experiments, 500 µL of the aqueous phase was always used, to which the extraction solvent was added in test volumes of 50, 250, or 500 µL. Subsequently, LLE was carried out according to the procedure described in the Section 3. When 500 µL of ESs was applied, an extraction efficiency (EE) > 92% was achieved for all BPs at the lowest 10 µg/mL level. The only exception is the most polar substance, BPS, which showed EE in the range of 87–92%. When only 50 µL of ES was used, the EE decreased to 65–98% for the most polar analytes, while for non-polar BPs such as BPC, BPG, and BPAP it remained >99%.

At the highest BP level of 40 µg/mL combined with the application of 500 µL ES, the EE for all analytes achieves values > 94%, again with the exception of polar BPs, for which it ranges between 93 and 97%. When applying 50 µL ES, the EE for the polar BPs decreased to 69–97%, while for the non-polar BPC, BPG, and BPAP, it remained at >94%.

**Figure 2:**
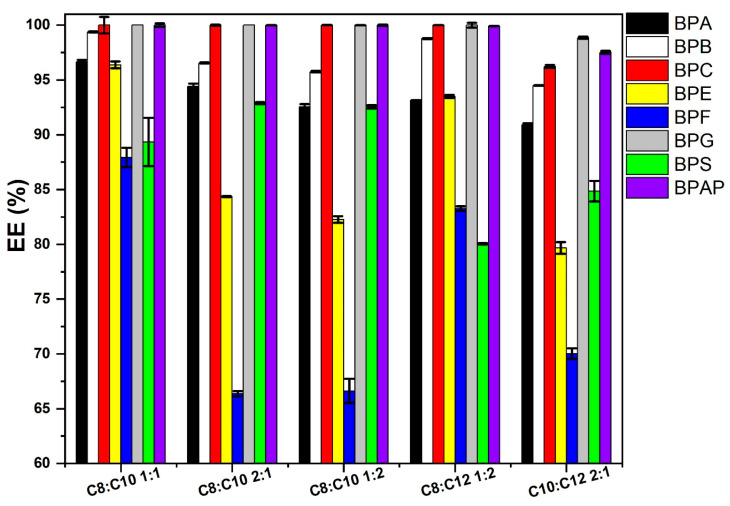
Comparison of EE for ESs based on a mixture of two carboxylic acids for removal of BPs from the aqueous phase at a level of 20 µg/mL, extraction solvent volume 50 µL, *n* = 3.

The EE for different ESs was compared for a BP level of 20 µg/mL. This level represents an ecologically relevant concentration and allows the detection of differences in the extraction power of individual solvents at a low ES application of 50 µL (Figure 2). Figure 2 indicates that the ES with a composition of C8:C10 1:1 exhibits the highest EE for all BPs except BPS. These LLE conditions were subsequently used to test other types of ESs.

#### 2.2.2. ES Based on Various Terpenes

These types of ESs contain terpineol, linalool, or geraniol in the HBA position and C8 in the HBD position, which are mixed in a 1:1 ratio. At a low level of BPs 10 µg/mL during extraction using 500 µL of solvent, EEs > 97% were achieved for all tested analytes; by reducing the volume of the extraction solvent to 50 µL, the EEs decreased to values > 90%. At high BP levels of 40 µg/mL using 500 µL ES, EEs reached values > 98%, and when 50 µL ES was applied, they ranged from 77 to 98%.

**Figure 3:**
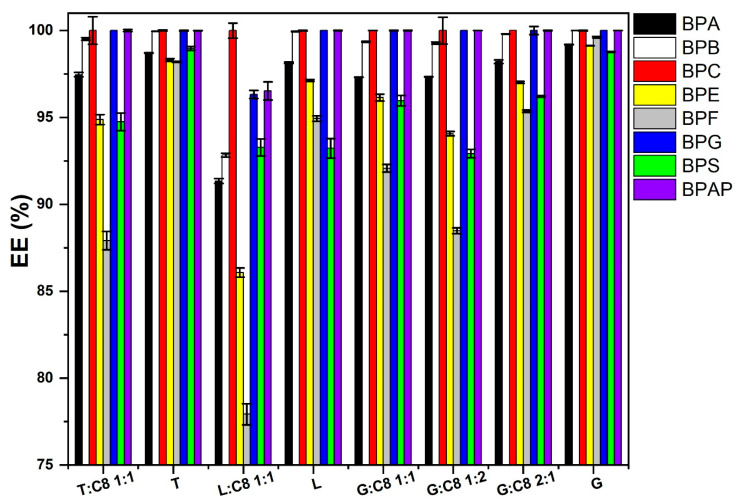
Comparison of EE for solvents based on various terpenes for removal of BPs from the aqueous phase at a level of 20 µg/mL, extraction solvent volume 50 µL, *n* = 3.

To compare terpene-based ESs, a 20 µg/mL level of BPs was used, and the results, summarized in Figure 3, show that the highest EE is exhibited by the equimolar mixture G:C8. Therefore, G:C8 mixtures in molar ratios of 2:1 and 1:2 were also prepared, which yielded similar EE values. For these reasons, G:C8 1:1 was selected for further studies, which is close to the recovery of individual terpenes such as terpineol, linalool, and geraniol. However, terpenes as pure solvents exhibit low *K*_ow_ values and are associated with relatively high solubility in the aqueous phase, which represents a significant obstacle to the extraction of BP from aqueous matrices.

#### 2.2.3. Menthol-Based ES

**Figure 4:**
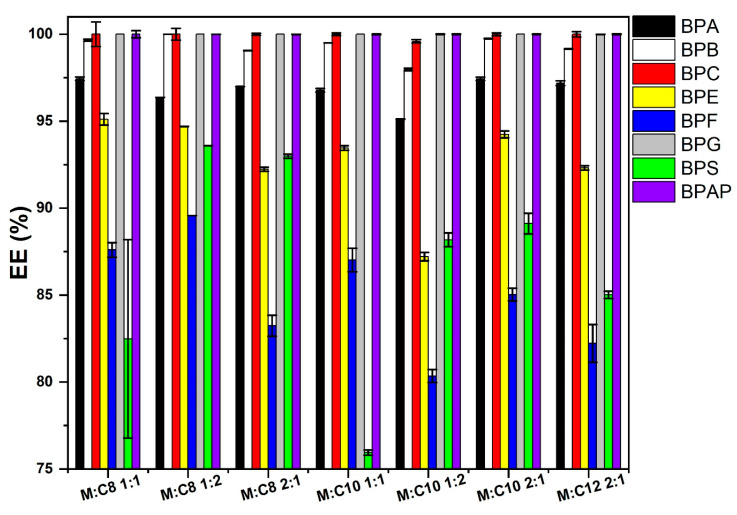
Comparison of EE for menthol-based ESs for removal of BPs from the aqueous phase at a level of 20 µg/mL, extraction solvent volume 50 µL, *n* = 3.

Menthol is generally considered a relatively inexpensive, natural, and environmentally friendly substance that is commonly used for preparation of ESs. Here, menthol is tested as HBA in combination with C8, C10, and C12 as HBD, and also in various molar ratios. At a BP concentration level of 10 µg/mL using 500 µL ES, EE > 93% was achieved for all analytes. When the ES volume was reduced to 50 µL, EE values ranged from 87 to 100% for polar BPs and were close to 100% for non-polar BPs. At a high level of 40 µg/mL and an extraction solvent volume of 500 µL, EE values were >98% for all analytes except the most polar BPS, for which they ranged between 93 and 98%. A comparison of ESs for LLE at a level of 20 µg/mL using 50 µL of extraction solvent shows that the highest EE is achieved with a mixture of M:C8 with a ratio of 1:2, as shown in Figure 4.

#### 2.2.4. Top-Rated ESs from Each Group and Optimization of LLE Conditions

**Figure 5:**
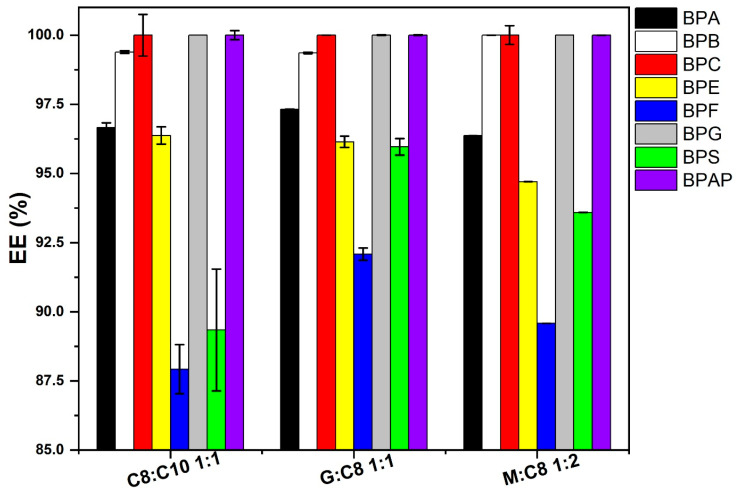
Comparison of EE for the best-rated ESs from each group for removal of BPs from the aqueous phase at a level of 20 µg/mL, extraction solvent volume 50 µL, *n* = 3.

Finally, the EEs achieved for the top-rated ESs from each group (C8:C10 1:1, G:C8 1:1, and M:C8 1:2) are compared in a single graph (Figure 5). The results show that all three selected ESs exhibit similar EEs and similar difficulties with LLE of more polar BPs, such as BPF or BPS. Nevertheless, all exhibit an EE > 85% across all analytes when using only 50 µL of extraction solvent. These representatives were then subjected to a more detailed analysis.

The effects of shaking time (5, 10, 15, and 20 min), shaking frequency (500, 1500, and 2500 rpm), and pH of the aqueous phase (2.0, 4.5, 7.0 and 8.0) adjusted with HCl and ammonia were tested. The experiments were performed for an equimolar mixture of BPs at a level of 20 µg/mL dissolved in 50 µL of the aqueous phase, and 50 µL of ES was used for extraction. The study shows that the highest EEs for most BPs were achieved after 15 min of shaking at a frequency of 1500 rpm. The pH of the aqueous phase has no effect on the LLE yield for most BPs. The only exceptions are the polar analytes BPE and BPF, for which it is necessary to suppress their dissociation, and a pH of 4.5 appears to be optimal.

### 2.3. Reuse of ESs and Their Absorption Capacity

**Figure 6:**
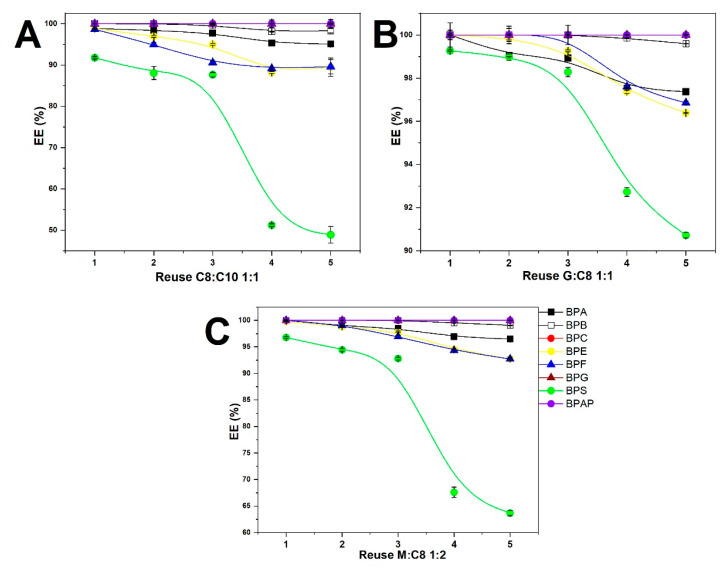
EEs for 5 cycles of LLE with repeated application of 500 µL ES for cleaning 50 µL of an aqueous mixture containing 8 BPs at a concentration of 20 µg/mL. (**A**) C8:C10 1:1, (**B**) G:C8 1:1, (**C**) M:C8 1:2. The EE values for BPC, BPG, and BPAP are nearly identical, approaching 100%. Therefore, the corresponding points overlap in the graph.

As the EE values for the three selected ESs reached values > 98% for all BPs except the BPS/C8:C10 1:1 combination, the repeated use of ES was tested, and the results are summarized in Figure 6. In this case, LLE experiments are based on the application of a large volume of 500 µL of solvent. Determining the absorption capacity of a solvent is an important environmental parameter and is of fundamental importance for its industrial use in wastewater treatment plants. However, when recycling solvents, it is necessary to take into account a gradual decrease in their effectiveness and to verify after how many cycles it is necessary to renew the pure reagent. In these experiments, 500 µL of solvent was applied to an LLE equimolar 20 µg/mL mixture of eight BPs dissolved in 50 µL of aqueous phase. After the first extraction was completed, the solvent was removed and reused for subsequent LLE of a new aqueous sample of BPs. A total of five repeating cycles were performed.

The results clearly show that the EE of solvents decreases with the number of cycles. The highest absorption capacity is exhibited by G:C8 1:1, where, after the fifth cycle, almost 100% EE is achieved for BPB, BPC, BPG, and BPAP, while for BPA, BPE, and BPF, EE drops to 97% and for BPS to 90%. In the case of purification by C8:C10 1:1 and M:C8 1:2, almost 100% extraction recovery is achieved for BPB, BPC, BPG, and BPAP after the fifth cycle, while for the other four BPs, the EE values are significantly worse than G:C8 1:1. The capacities of the solvents thus decreases in the order G:C8 1:1 > C8:C10 1:1 > M:C8 1:2.

A very important factor in the process of removing pollutants from aqueous matrices is the regeneration of the depleted ES as an extraction agent and the achievement of a closed purification cycle. Simple regeneration of the ES is based on the evaporation of pollutants, a process applicable only to volatile substances such as polyaromatic hydrocarbons [23]. Adsorption of pollutants onto activated carbon is also used, where the sorbent is added directly to the ES and the ES is subsequently regenerated by filtration [22]. In the case of phenolic substances such as BPs, alkalization of the ES by adding NaOH is an option [35]. Through this process, the BPs concentrated in the ES are converted into sodium bisphenolates, which are insoluble in the hydrophobic ES, and subsequently re-extracted into the aqueous phase.

### 2.4. Real Samples

The developed methodology was next applied to determine BPs in real samples. Industrial samples with a high probability of BP occurrence were selected for analysis, such as polycarbonate plastics, plastic water pipes, and thermal paper receipts (Table 4).

**Figure 7:**
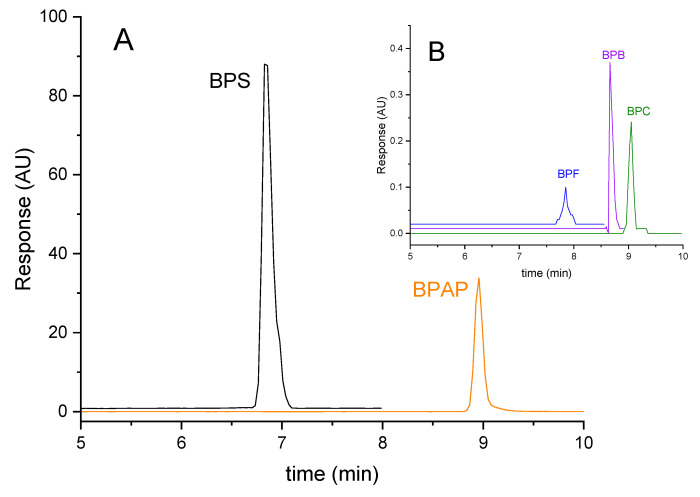
HPLC-MS/MS analysis of an aqueous extract from the thermal paper, with MRM records of dominant (**A**) and minor (**B**) BPs. Experimental conditions are as in Figure 1.

Only BPA was found in quantifiable amounts in the aqueous extract of polycarbonate. On the other hand, five BPs were determined in the aqueous extract from thermal paper, including PBS and BPAP in high concentrations. The absence of BPA and the high concentration of BPS are consistent with the fact that BPA is being replaced by BPS, particularly in the recycling of paper products [36]. Surprisingly, the highest concentration was found for BPAP, which was not detected in the other samples. In contrast, BP levels in the aqueous extract of the water pipe were below the LOQ of the developed HPLC-MS/MS method. The results indicate that the extraction efficiency of all the tested EEs is sufficient for 100% removal of PBs found in real samples. Figure 7 shows the HPLC-MS/MS analysis of an aqueous extract from thermal paper.

The analytical performance of the proposed method, including limits of detection (LODs), limits of quantification (LOQs), and extraction recovery, is comparable to that of the current method (see Table 5). The pentafluorophenyl-based stationary phase enables the separation of the eight bisphenols under study in 13 min. It thus provides complementary selectivity to the already established C18 stationary phases.

## 3. Experimental Methodology

### 3.1. Chemicals and Solutions

The following chemicals were used to prepare standard solutions of BPs: BPA (≥99%, Sigma Aldrich, Taipei, Taiwan), BPB (≥98%, Sigma Aldrich, Shanghai, China), BPC (≥99%, Sigma Aldrich, St. Louis, MO, USA), BPE (≥98%, Sigma Aldrich, China), BPF (≥98%, Sigma Aldrich, Tokyo, Japan), BPG (≥98%, Sigma Aldrich, Oakville, ON, Canada), BPS (≥98%, Sigma Aldrich, China), BPAP (≥99%, Sigma Aldrich, Japan), BPA diphenyl 13C12 (98%, Sigma Aldrich, USA) as internal standard (IS), EtOH (96%, Lach-Ner, Neratovice, Czech Republic) and DEI as solvents. All stock solutions of BPs were stored in a refrigerator at 5 °C. Calibration solutions of BPs were prepared by diluting 1 mg/mL ethanolic stock solutions with DEI. The concentrations of BPs calibration solutions in deionized (DEI) water were: 1, 10, and 200 ng/mL and 1, 5, 10, 15, 20, and 25 µg/mL. IS at a concentration of 10 µg/mL was added to each calibration solution.

To prepare mobile phases for HPLC and eliminate carry-over effects, the following substances were used: MeOH (hypergrade for LC-MS LiChrosolv, Supelco, Sigma Aldrich, Darmstadt, Germany), ammonia (25%, Lach-Ner, Czech Republic), acetonitrile (≥99.9%, LC-MS Chromasolv, Honeywell Riedel-de Haën, Seelze, Germany), DMSO (Lachema, Brno, Czech Republic) and DEI.

The following chemicals were used to prepare the extraction solvents and ESs: caprylic acid (C8, ≥98%, Fluka, Kelana Jaya, Malaysia), capric acid (C10, ≥98%, Sigma Aldrich, Germany), lauric acid (C12, ≥99%, Sigma Aldrich, Petaling Jaya, Malaysia), myristic acid (C14, ≥99%, Merck, Petaling Jaya, Malaysia), terpineol (T, mixture of isomers, Sigma Aldrich, Saint-Quentin-Fallavier, France), linalool (L, 97%, Sigma Aldrich, China), geraniol (G, 98%, Sigma Aldrich, USA), DL-menthol (M, 99%, Sigma Aldrich, China). The pH of solvents and mobile phases was adjusted using HCl (35%, Lach-Ner, Czech Republic) and ammonia (25%, Lach-Ner, Czech Republic). DEI water used for the preparation of the aqueous solutions was purified using a Milli-Q water purification system (Millipore, Bedford, MA, USA).

### 3.2. Instrumentation for HPLC/MS

Chromatographic analyses were performed on a high-performance liquid chromatograph Agilent 1290 Infinity (Agilent Technologies, Waldbronn, Germany), which consisted of the following parts: high-pressure pump (Agilent 1290 Infinity), thermostatically controlled automatic dispenser (Agilent 1290 Infinity), and column thermostat (Agilent 1290 Infinity). An Agilent 6460 Triple Quadrupole LC/MS System (Agilent Technologies, Germany) with electrospray ionization was used for detection. The measured data were processed using Agilent Masshunter Workstation Software, version B.01.03 (Agilent Technologies) and Microsoft Excel (Microsoft Corporation, Redmond, WA, USA). Chromatographic analyses were performed on an Ascentis Express F5 column (150 × 4.6 mm, 2.7 µm, Supelco, Bellefonte, PA, USA). Statistical data processing and graphical representation were performed in Microsoft Excel 2016 (Microsoft Corporation) and Origin 2023 (OriginPro Corporation, Northampton, MA, USA). An APX-100 analytical balance (Denver Instrument, Passau, Germany) was used for weighing analytes, internal standards, and real samples.

### 3.3. Preparation of Eutectic Solvents and LLE Procedure

The binary ESs were always prepared by mixing precisely weighed amounts of HBA and HBD in molar ratios of 1:1, 1:2, or 2:1. The mixture was then heated in a glass vial at 85 °C and stirred with a magnetic stirrer (Arex Digital Heating Magnetic Stirrer, Velp Scientifica, Usmate Velate MB, Usmate Velate, Italy) at a frequency of 150 rpm for a minimum of 15 min until a clear solvent was formed. A total of 18 ESs with different HBA/HBD compositions or different molar ratios (*n*:*n*) of both components were prepared in this way, as shown in Table 6. The resulting ES was tempered to laboratory temperature before use for LLE. In addition, pure solvents such as terpineol, linalool, and geraniol were also tested for extraction. The water content in the prepared ESs after tempering to 25 °C was determined by Karl Fischer titration using an 831 KF Coulometer (Metrohm, Herisan, Switzerland), as shown in Table 6. The measured data show that the water content in ES decreases with the length of the carboxylic acid chain used. However, no correlation was found between the water content in the ES and the LLE recovery for BPs.

LLE of model aqueous samples of BPs was performed at a controlled temperature of 25.0 ± 0.1 °C in a 1 mL Eppendorf tube. Always, 500 μL of aqueous sample spiked with an equimolar mixture of BPs at three concentration levels of 10, 20 and 40 μg/mL was used for extraction. A precise volume of ES was added to this aqueous sample (see Section 2 for details). The mixture was then shaken (Multi Speed Vortex instrument, MSV-3500, Biosan, Riga, Latvia) for 15 min at a frequency of 1500 rpm (these LLE conditions were taken from a previous study [41,42]). Fifteen minutes of shaking represents the minimum time required to achieve equilibrium, after which the mixture was centrifuged at 2500 rpm for 5 min to complete separation of the aqueous and organic phases. Then, 150 μL of aqueous phase was collected, mixed with IS solution with a final concentration of 10 µg/mL, and the prepared sample was analyzed using the HPLC-MS/MS method. Each LLE was performed three times, and the aqueous sample obtained was measured three times for BP content by HPLC-MS/MS. LOD and LOQ were calculated based on the SD for a six-times repeated analysis of the blank sample and the slope of the calibration curve for peak height, LOD = 3 SD/slope, LOQ = 10 SD/slope

### 3.4. Preparation of Real Samples

For the extraction of BPs from real samples, an accurate weight of dry sample was always used, DEI was added, and the sample was heated at 40 °C for 24 h in a beaker covered with parafilm. The aqueous matrix was then filtered, and the resulting extract was evaporated at 100 °C to the final volume. Then, 150 μL of sample was mixed with IS solution with a final concentration of 10 µg/mL, and the sample was subjected to HPLC-MS/MS determination. All samples were measured in triplicate. All samples were measured three times, and Table 4 lists the average concentrations of the individual PBs found in the samples. Details of the processing of individual types of real matrices are given in Table 7.

## 4. Conclusions

ESs have been shown to be effective extraction agents for removing BPs from aqueous matrices. A comparison of three types of binary mixtures, such as organic acid/organic acid, terpene/organic acid, and menthol/organic acid, revealed that the highest extraction yield was achieved with a mixture of geraniol:caprylic acid prepared in a molar ratio of 1:1. This ES can be used repeatedly for up to five consecutive cycles, and almost 100% recovery is achieved for more hydrophobic types such as BPB, BPC, BPG, and BPAP, while for more polar types such as BPA, BPE, and BPF, the yield drops to 97% and for BPS to 90%. Therefore, this binary mixture is potentially promising and environmentally friendly for wastewater treatment. Furthermore, it appears that the pentafluorophenyl-based stationary phase ensures the separation of the eight BPs under investigation within 13 min, thereby providing selectivity comparable to that of conventional C18.

## Figures and Tables

**Table 1:** Main types of BPs and their physical and chemical properties.

BPs	Abbreviation	*M*_r_	log *K*_ow_	p*K*_A_	*S*_w_, mg/L
2,2-bis(4-hydroxyphenyl)propane	BPA	228.3	3.43	10.1	120.0
2,2-bis(4-hydroxyphenyl)butane	BPB	242.3	4.13	10.1	29.2
2,2-bis(4-hydroxy-3-methylphenyl)propane	BPC	256.3	4.33 *	10.4 *	-
1,1-bis(4-hydroxyphenyl)ethane	BPE	214.3	2.98 *	10.1 *	265.0
4,4’-dihydroxydiphenylmethane	BPF	200.2	2.91	9.9 *	408.1
2,2-bis(4-hydroxy-3-isopropylphenyl)propane	BPG	312.5	5.62 *	10.4 *	-
4,4’-sulphonyldiphenol	BPS	250.3	1.65	8.2	3518.0
4,4’-(1-phenylethyliden)bisphenol	BPAP	290.4	4.86	10.2 *	3.8

* predicted values taken from SciFinder, *K*_ow_—octanol/water partition coefficient, *S*_w_—water solubility.

**Table 2:** Optimized conditions of tandem mass spectrometric detection for BPs and IS.

Analyte	Precursor Ion, *m*/*z*	Product Ion, *m*/*z*	Confirmation Ion, *m*/*z*	Voltage on Fragmentor, V	Collision Energy, V
BPA	227.1	212.1	133.1	135	15
BPB	241.1	211.1	147.2	110	30
BPC	255.2	147.2	239.1	115	30
BPE	213.1	197.8	119.2	100	15
BPF	199.1	93.1	105.1	130	15
BPG	311.2	295.2	175.2	120	30
BPS	249.0	108.0	91.8	120	30
BPAP	289.2	274.1	194.9	120	20
IS	239.1	139.1	-	120	30

**Table 3:** Parameters of regression equations for peak areas, coefficient of determination, LOD, LOQ, and linear dynamic range (LDR) for calibration of model samples of BPs in DEI; SD values in parentheses.

Analyte	Regression Equation	*R* ^2^	LOD, ng/mL	LOQ, ng/mL	LDR, ng/mL
BPA	y = 0.3 (0.012) x − 0.06 (0.002)	0.9995	8.75	29.1	29.1–10,000
BPB	y = 0.7 (0.009) x + 0.07 (0.001)	0.9978	3.25	10.8	10.8–5000
BPC	y = 0.2 (0.003) x + 0.03 (0.001)	0.9947	3.03	10.1	10.1–5000
BPE	y = 1.4 (0.049) x + 0.09 (0.005)	0.9976	1.01	3.33	3.3–5000
BPF	y = 0.3 (0.008) x + 0.06 (0.004)	0.9913	3.10	10.2	10.2–10,000
BPG	y = 0.4 (0.012) x + 0.05 (0.003)	0.9987	4.14	13.8	13.8–5000
BPS	y = 15 (1.6) x + 4.9 (0.3)	0.9989	0.07	0.24	0.24–1000
BPAP	y = 2.9 (0.16) x − 0.41 (0.04)	0.9924	1.12	3.75	3.8–5000

**Table 4:** The content of individual bisphenols found in the tested real samples in ng/mL.

Analyte	Polycarbonate	Water Pipe	Thermal Paper
BPA	553	<LOQ	-
BPB	-	-	66
BPC	-	-	145
BPE	-	-	-
BPF	-	-	32
BPG	-	-	-
BPS	<LOQ	<LOQ	2195
BPAP	-	-	3295

**Table 5:** Comparison of the presented method with other protocols for the extraction and analysis of BPs in real samples.

Analyte	Extraction	Samples	EE, %	Control Method/LOD	SF/Run Time	Ref.
17 BPs	-	Canned food, drinkware	-	LC-MS/MS/0.7–10.8 ng/L	C18/8 min	[26]
BPA, BPB, BPF, BPP, BPS, BPZ, BPAF, BPAP	ACN + 1% AcOH	Dairy products	88.2–108	LC-MS/MS/0.8–200 mg/kg	C18/15 min	[27]
BPA, BPB, BPS	TBAB, M/organic acids	Canned fruit	79.5–101	UPLC-MS/MS/1.5– 3.0 ng/g	C18/13 min	[37]
BPA, BPB, BPE, BPF, BPS	C18 SPE	Canned soft drinks	85–100	LC-MS/MS/5–50 ng/L	C18/3 min	[38]
BPA, BPS, BPE, BPF, BPAP	imprinted SPE	Sewage, sludge	44–101	HPLC-MS/MS/0.0007–16.3 ng/L	C18/30 min	[25]
BPA	M/vaniline; M/*trans*-cinnamic acid	Herbal aqueous samples	86–115	Nano LC-UV/34–125 mg/mL	C30/18 min	[39]
BPA	M/organic acids	Water environment	78–98	HPLC–UV/12 µg/mL	C18/4 min	[40]
BPS		Thermal paper		HPLC-MS/MS/7 pg/mL	C18/15 min	[36]
BPA, BPB, BPC, BPE, BPF, BPG, BPS, BPAP	terpenes/organic acids	Polycarbonate, water pipe, thermal paper	78–100	HPLC-MS/MS/0.07–8.75 ng/mL	F5/13 min	Present work

TBAB, tetrabutylammonium bromide; F5, pentafluorophenylpropyl.

**Table 6:** Overview of prepared ESs with specifications of HBA and HBD composition, molar ratio, and water content determined according to Karl Fischer.

HBA	HBD	Abbreviation	Molar Ratio	Water Content, ppm
Caprylic acid	Capric acid	C8:C10	1:1	532
Caprylic acid	Capric acid	C8:C10	1:2	357
Caprylic acid	Capric acid	C8:C10	2:1	592
Caprylic acid	Lauric acid	C8:C12	2:1	511
Capric acid	Lauric acid	C10:C12	2:1	275
Terpineol	Caprylic acid	T:C8	1:1	1297
Linalool	Caprylic acid	L:C8	1:1	622
Geraniol	Caprylic acid	G:C8	1:1	8500
Geraniol	Caprylic acid	G:C8	1:2	10,953
Geraniol	Caprylic acid	G:C8	2:1	15,242
DL-menthol	Caprylic acid	M:C8	1:1	1108
DL-menthol	Caprylic acid	M:C8	1:2	1100
DL-menthol	Caprylic acid	M:C8	2:1	755
DL-menthol	Capric acid	M:C10	1:1	773
DL-menthol	Capric acid	M:C10	1:2	671
DL-menthol	Capric acid	M:C10	2:1	691
DL-menthol	Lauric acid	M:C12	2:1	493
DL-menthol	Myristic acid	M:C14	2:1	-
Terpineol	-	T	-	1758
Linalool	-	L	-	464
Geraniol	-	G	-	14,891

**Table 7:** Real industrial samples containing BPs and details of their laboratory processing.

Sample	Weight, g	DEI Volume, mL	Final Volume, mL
Polycarbonate	1.5	10.0	3.0
Water pipe	6.0	6.0	2.0
Thermal paper	1.5	60.0	5.0

## Data Availability

The raw data supporting the conclusions of this article will be made available by the authors on request.
